# Proteomics profile in encapsulated follicular patterned thyroid neoplasms

**DOI:** 10.1038/s41598-024-67079-6

**Published:** 2024-07-16

**Authors:** Truong Phan-Xuan Nguyen, Sittiruk Roytrakul, Supranee Buranapraditkun, Shanop Shuangshoti, Nakarin Kitkumthorn, Somboon Keelawat

**Affiliations:** 1 Department of Pathology, Faculty of Medicine, Chulalongkorn University, Bangkok, 10330 Thailand; 2 Functional Proteomics Technology Laboratory, National Center for Genetic Engineering and Biotechnology, National Science and Technology Development Agency, Pathumthani 12120, Thailand; 3 Division of Allergy and Clinical Immunology, Department of Medicine, King Chulalongkorn Memorial Hospital, Faculty of Medicine, Chulalongkorn University, Thai Red Cross Society, Bangkok, 10330 Thailand; 4 Center of Excellence in Thai Pediatric Gastroenterology, Hepatology and Immunology (TPGHAI), Faculty of Medicine, Chulalongkorn University, Bangkok, 10330 Thailand; 5 Chulalongkorn GenePRO Center, Faculty of Medicine, Chulalongkorn University, Bangkok, 10330 Thailand; 6 Department of Oral Biology, Faculty of Dentistry, Mahidol University, Bangkok, 10330 Thailand; 7 Precision Pathology of Neoplasia Research Group, Department of Pathology, Faculty of Medicine, Chulalongkorn University, Bangkok, 10330 Thailand

**Keywords:** Follicular-patterned thyroid tumors, Invasive Encapsulated Follicular Variant of Papillary Thyroid Carcinoma, Non-invasive Follicular Thyroid Neoplasm with Papillary-like Nuclear Features, Well-Differentiated Tumor of Uncertain Malignant Potential, Proteomics, Mass spectrometry, Molecular medicine, Oncology, Pathogenesis

## Abstract

Diagnosing encapsulated follicular-patterned thyroid tumors like Invasive Encapsulated Follicular Variant of Papillary Thyroid Carcinoma (IEFVPTC), Non-invasive Follicular Thyroid Neoplasm with Papillary-like Nuclear Features (NIFTP), and Well-Differentiated Tumor of Uncertain Malignant Potential (WDT-UMP) remains challenging due to their morphological and molecular similarities. This study aimed to investigate the protein distinctions among these three thyroid tumors and discover biological tumorigenesis through proteomic analysis. We employed total shotgun proteome analysis allowing to discover the quantitative expression of over 1398 proteins from 12 normal thyroid tissues, 13 IEFVPTC, 11 NIFTP, and 10 WDT-UMP. Principal component analysis revealed a distinct separation of IEFVPTC and normal tissue samples, distinguishing them from the low-risk tumor group (NIFTP and WDT-UMP). IEFVPTC exhibited the highest number of differentially expressed proteins (DEPs) compared to the other tumors. No discriminatory proteins between NIFTP and WDT-UMP were identified. Moreover, DEPs in IEFVPTC were significantly associated with thyroid tumor progression pathways. Certain hub genes linked to the response of immune checkpoint inhibitor therapy, revealing the potential predictor of prognosis. In conclusion, the proteomic profile of IEFVPTC differs from that of low-risk tumors. These findings may provide valuable insights into tumor biology and offer a basis for developing novel therapeutic strategies for follicular-patterned thyroid neoplasms.

## Introduction

Thyroid cancer is the most prevalent endocrine malignancy, ranking the tenth among cancers in both sexes and the fifth among female cancers. It accounts for 1–2% of all new cancer cases diagnosed annually worldwide^1,2^. The latest version of the 5th World Health Organization's Classification of Endocrine and Neuroendocrine Tumors has redefined thyroid cancers, providing insights into their biology, histogenesis, and molecular profiles^3,4^. Certain thyroid follicular lesions present diagnostic challenges due to morphological and architectural similarities, leading to interobserver variations among practitioners in the field of thyroid pathology^5^. Encapsulated follicular-patterned tumors share predominant follicular patterns (over 95%) and are surrounded by thin or thick fibrous capsules^4,6,7^. This category encompasses non-invasive follicular thyroid neoplasm with papillary-like nuclear characteristics (NIFTP), well-differentiated tumors with unknown malignant potential (WDT-UMP), and invasive encapsulated follicular variant of papillary thyroid carcinoma (IEFVPTC). NIFTP lacks signs of capsular or vascular invasion, in contrast to IEFVPTC that clearly exhibits such findings. WDT-UMP displays ambiguous pattern regarding capsular or vascular invasion^8,9^. These follicular-patterned thyroid neoplasms often exhibit *RAS*-like molecular alterations and the absence of *BRAF* V600E. It also encompasses a range from low-risk neoplasms to malignant neoplasms. The recent WHO classification introduced "differentiated high-grade thyroid carcinoma," categorized as high-grade PTC, high-grade FTC, or high-grade oncocytic carcinoma of the thyroid (OCA), based on mitotic activity (≥ 5 per 2 mm^2^) and/or the presence of necrosis, broadening the range of tumors in this group ^10^. In the context of thyroid cancer management, there is a growing inclination towards personalized medicine to prevent overdiagnosis and overtreatment. Understanding biological processes in various thyroid lesions aids in diagnosis and treatment decisions.

Liquid chromatography tandem mass spectrometry (LC–MS/MS) presents a promising approach for the identification and quantification of numerous proteins in biological samples^11^. This proteomic workflow is available to analyze proteins, detect post-translational modifications, compare protein abundance in different sample sets (e.g., healthy individuals vs. diseased patients, various tumor subtypes or stages), and explore protein interactions and complexes within cellular pathways or networks. These analyses aim to deepen our comprehension of biological processes, unravel underlying mechanisms, enhance disease diagnosis and prognosis, and facilitate precise patient stratification for personalized medicine^11^. LC–MS/MS is highly advantageous, reliable, robust, and sensitive in achieving these objectives. This method has been utilized in several studies to identify potential biomarker candidates that could differentiate between follicular-patterned thyroid tumors^12,13^. Nevertheless, the different proteomic variations of encapsulated follicular-patterned thyroid tumors, such as NIFTP, WDT-UMP, and IEFVPTC, remain unvalidated. Through this approach, we aimed to discern the similarities and differences in protein levels among these three types of thyroid tumors.

## Results

### Patient characteristics and study design

**Figure 1:**
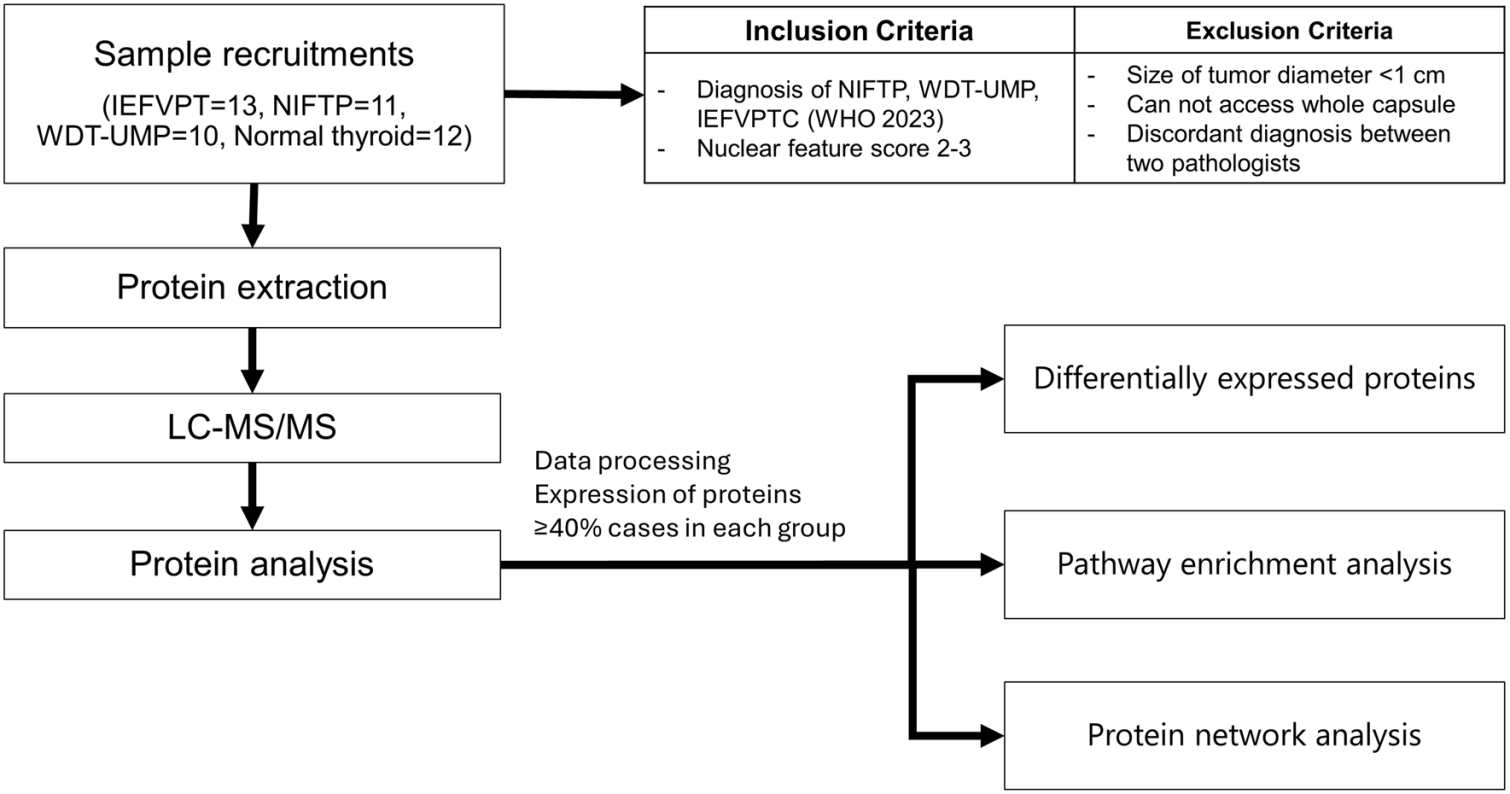
Workflow of the current study.

**Figure 2:**
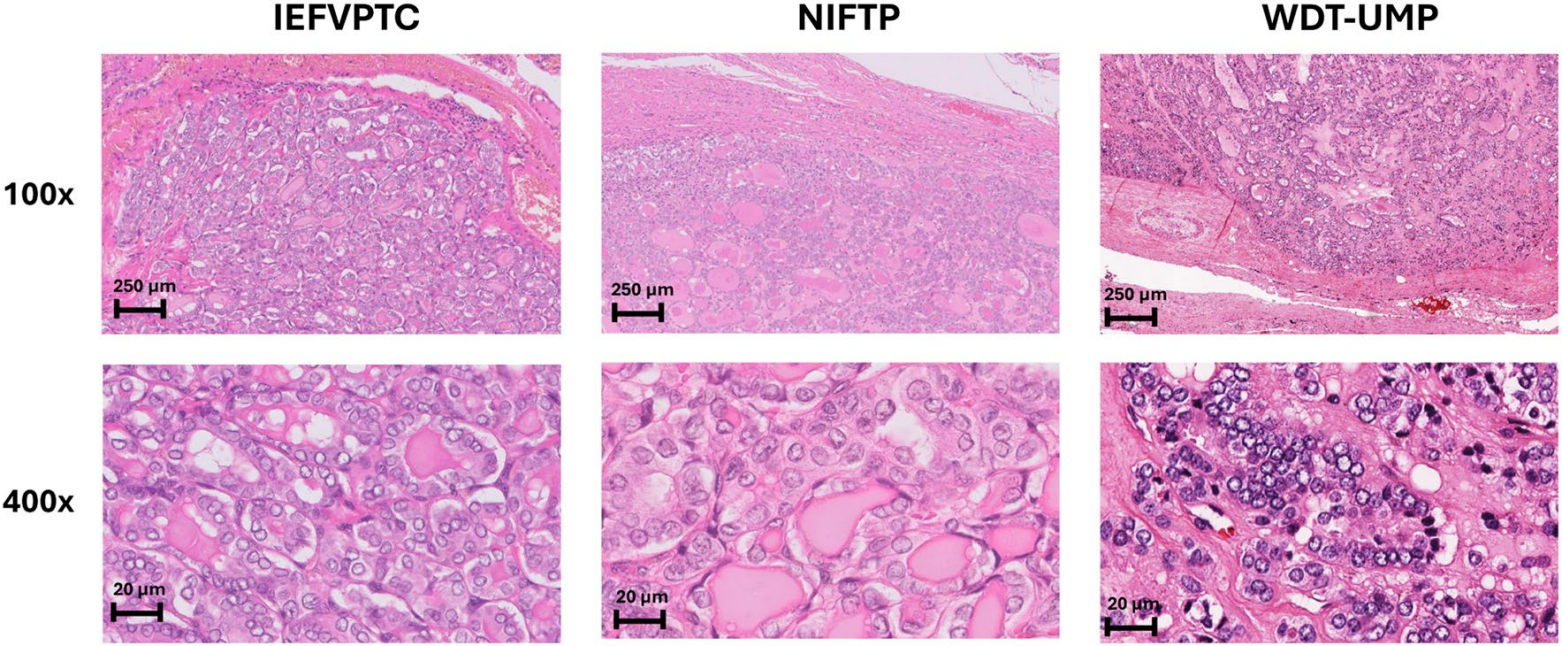
The histopathological characteristics of IEFVPTC, NIFTP, and WDT-UMP. These tumors have the same nuclear characteristics as papillary thyroid cancer and are surrounded by a thin or thick fibrous capsule. NIFTP lacks signs of capsular or vascular invasion, whereas IEFVPTC clearly demonstrates such invasion. WDT-UMP have uncertain capsular or vascular invasion.

In this study, we assembled a collection of 46 specimens for proteomic analysis. The samples were processed according to the procedures outlined in the “Methods” section (Fig. 1). Further details regarding the clinical characteristics were summarized in Table 1 and Supplementary Table 1. Across the four groups, there were no statistically significant differences observed in terms of age, nodule size, and nuclear score. The median patient age was 43 years, with a range of 24–79 years, and there was a predominance of female patients. The average size of tumor nodules ranged from 30 to 40 mm in diameter. Histopathological features were illustrated in Fig. 2. All tumors had a nuclear score of 2–3, mitotic count is < 3 per 2 mm^2^ and no necrosis was detected. IEFVPTC had a fibrous capsule or well-defined border, frequently exhibited capsular invasion (8 minimally invasive cases, 1 widely invasive cases) or 1–3 foci vascular invasion (4 encapsulated angioinvasive cases). NIFTP were well-encapsulated thyroid nodules and < 1% papillae. WDT-UMP is encapsulated or unencapsulated but well-circumscribed, in which invasion remains questionable after thorough sampling and exhaustive examination.

**Table 1:** Clinicopathological characteristics of patients.

Characteristic	IEFVPTC (n = 13)	NIFTP (n = 11)	WDT-UMP (n = 10)	Normal thyroid (n = 12)	P-value
Age (year)					P > 0.05
Median	52	47	40.5	42	
Range	27–70	36–70	24–79	24–70	
Gender	3.3:1	2.6:1	1.5:1	1:1	
Female: Male ratio					
Nodule size (diameter—mm)				N/A	P > 0.05
Mean	40.38	30.55	34.9		
Range	22–70	15–60	15–84		
Nuclear score (mean)	2.77	2.73	2.8	N/A	P > 0.05

IEFVPTC, invasive encapsulated follicular variant of papillary thyroid carcinoma; NIFTP, noninvasive follicular thyroid neoplasm with papillary-like nuclear features; WDT-UMP, Well differentiated thyroid tumor of uncertain malignant potential.

### Differentially Expressed Protein (DEPs) of the encapsulated follicular-patterned thyroid tumors

**Figure 3:**
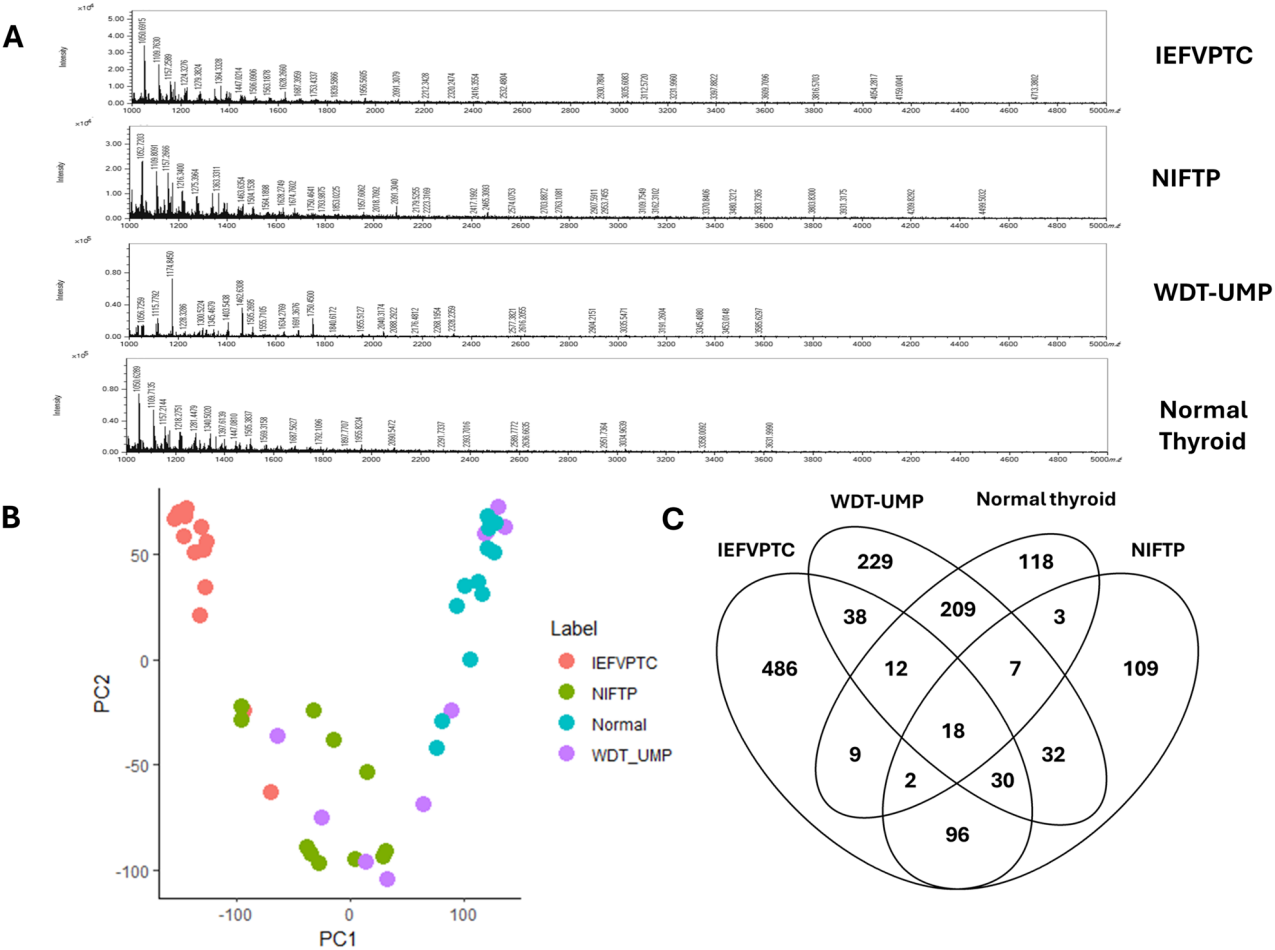
Global proteomic analysis. (**A**) The average spectra of each group in the range of 1000–5000 m/z. (**B**) Principal component analysis (PCA) using 1398 proteins grouped by tissue types. (**C**) Venn diagram showing protein identification in IEFVPTC, NIFTP, WDT-UMP and normal.

After preprocessing and filtering to ensure the inclusion of proteins presented in more than 40% frequency of samples in each group, we identified a total of 1398 proteins from the 46 proteomic data files. The figures illustrated the numbers of identified protein in each MS file (Supplementary Fig. 1 A-B). Notably, there is a disparity among the four groups in mass spectrum chart, with IEFVPTC and NIFPT displaying a higher molecular mass/charge exceeding 3000 (Fig. 3A). Principal component analysis using 1398 proteins categorized by tissue type (Fig. 3B) indicated a distinct separation of IEFVPTC and normal tissue samples which were two densely-packed groups in the upper area of the graph, NIFPT intermix with WDT-UMP, a low-risk tumor group. Consequently, there were distinct protein expression profiles across normal thyroid tissue, IEFVPTC and low-risk tumors (NIFTP and WDT-UMP). The Venn diagram (Fig. 3C) illustrated the number of identified proteins exhibiting quantitative similarities and differences among the four groups, with 486, 109, 229, and 118 proteins specifically identified in IEFVPTC, NIFTP, WDT-UMP, and normal thyroid, respectively.

**Figure 4:**
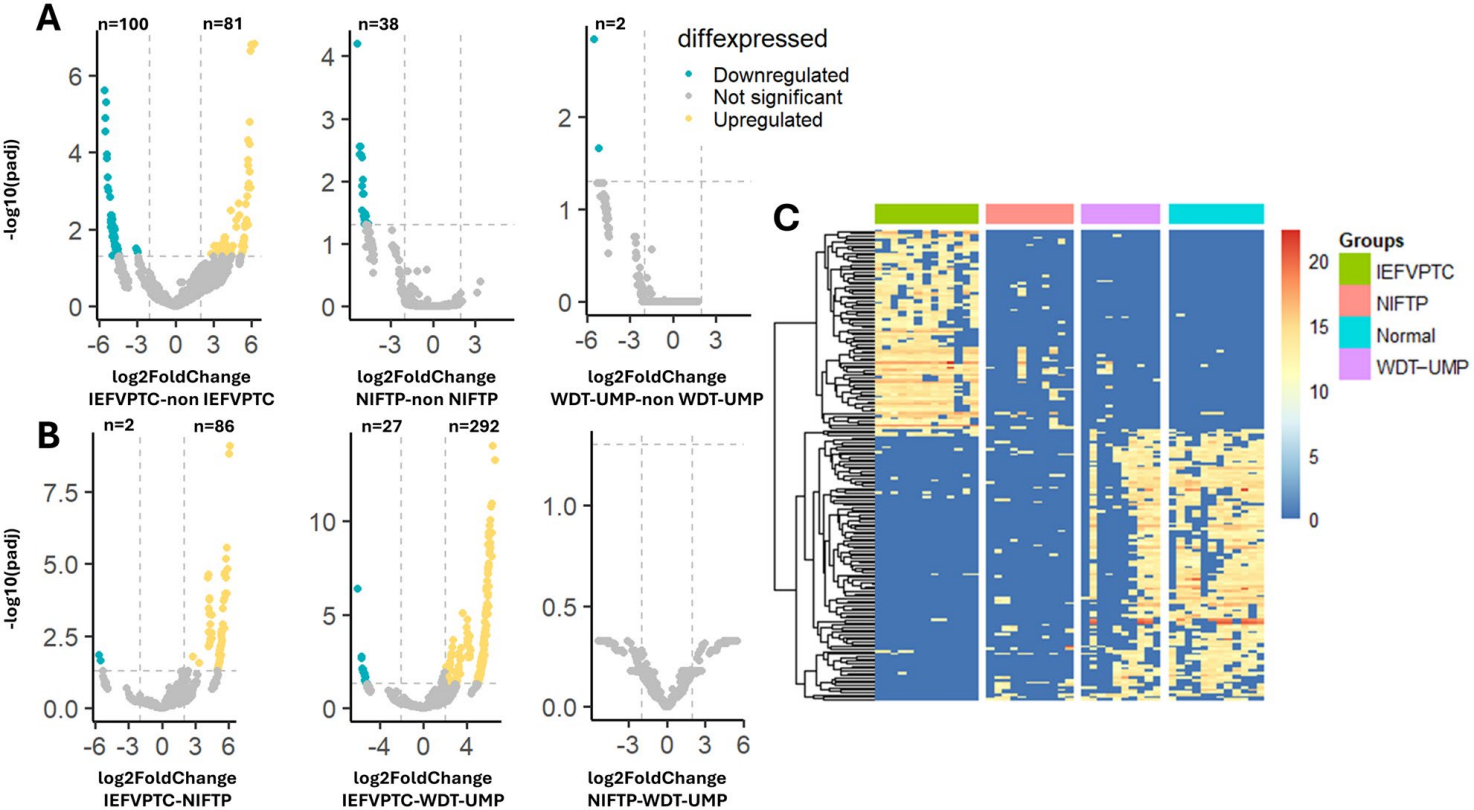
Differential protein expression analysis. (**A**) Volcano plot showing differential proteins in IEFVPTC vs. non-IEFVPTC, NIFTPC vs. non-NIFTP, WDT-UMP vs. non-WDT-UMP and (**B**) IEFVPTC vs. NIFTP, IEFVPTC vs. WDT-UMP, NIFTP vs. WDT-UMP with a two-fold-change cutoff and an adjusted P value threshold less than 0.05. A heatmap was generated to depict the protein expression profiles of specimens from the four groups. Proteins (rows) were clustered without supervision, and samples (columns) were organized based on tissue type. The color gradients on the heatmap represent the intensity of each protein in the respective samples.

To explore similarities and differences among IEFVPTC, NIFTP, and WDT-UMP samples, we compared the differential expression of multiple proteins. Volcano plots (Fig. 4A) revealed 181 DEPs between IEFVPTC and non-IEFVPTC, with 100 upregulated and 81 downregulated proteins identified. Among the top 10 significant DEPs, six were upregulated (*NPAP1, STK33, O51I2, DLG4, THUM2, CODA1*), and four were downregulated (*CCHCR, STAR5, DDX6, LIGO2*) (Supplementary Table 2). Additionally, 38 downregulated proteins were observed in NIFTP compared to non-NIFTP (Supplementary Table 3). Interestingly, only two downregulated proteins (TMCC1, ZN540) were found between WDT-UMP and non-WDT-UMP, a lower number than in other comparative analyses (Supplementary Table 4).

Pairwise comparisons of the differential expression of multiple proteins in the IEFVPT, NIFTP and WDT-UMP samples were also performed (Fig. 4B). IEFVPTC had the most DEPs when compared to NIFTP and WDT-UMP which expressed 86 upregulated and 2 downregulated proteins, 292 upregulated and 27 downregulated proteins, respectively. Interestingly, no discriminatory proteins were detected between NIFTP and WDT-UMP (Supplementary Tables 5, 6).

These analyses of protein expression highlighted a distinct separation of IEFVPTC from NIFTP, WDT-UMP, and normal tissue, indicating that malignant tumors exhibit distinct protein profiles compared to low-risk tumors group. Whereas NIFTP showed no apparent distinction from WDT-UMP. Subsequently, an unsupervised clustering protein heatmap (Fig. 4C) was generated using 196 discriminatory proteins, indicating higher abundance in malignant tumor, and facilitating the distinction of IEFVPTC from NIFTP and WDT-UMP.

### Biological analysis for IEFVPTC

While IEFVPTC, NIFTP, and WDT-UMP belong to the category of encapsulated follicular-pattern thyroid tumors and share a *RAS*-like molecular profile, IEFVPTC stands out as malignant and demonstrates a greater diversity in proteins compared to the others. This was the reason why we aimed to concentrate on identifying central genes, critical pathways, and potential molecular mechanisms. These findings could have significant clinical implications for the treatment and diagnosis of IEFVPTC.

**Figure 5:**
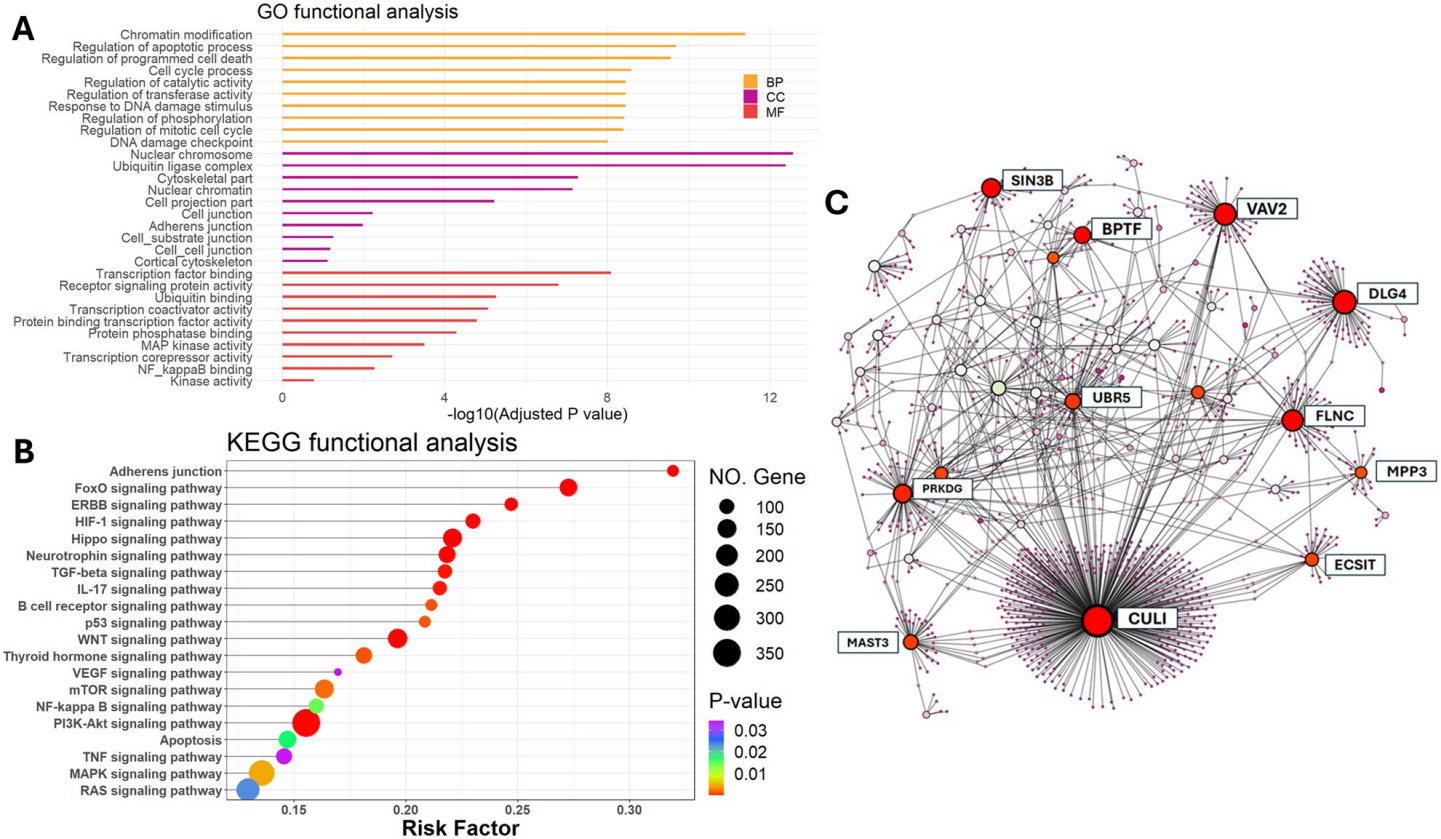
Biological analysis of IEFVPTC (**A**) GO analysis of the significant DEPs of IEFVPTC. (**B**) KEGG pathway analysis significant DEPs of IEFVPTC. (**C**) The PPI of DEPs through NetworkAnalyst showing network for CUL1, PRKDC, DLG4, VAV2, FLNC, UBR5, MAST3, SIN3B proteins. GO, Gene Ontology; BP, biological process; CC, cellular component; MF, molecular function; KEGG, Kyoto Encyclopaedia of Genes and Genomes; PPI, protein–protein interaction.

Utilizing NetworkAnalyst, we performed a GO biological function enrichment analysis for the 181 significant DEPs associated with IEFVPTC. In terms of biological processes, these proteins were predominantly linked to chromatin modification, the regulation of apoptotic processes, cell cycle processes, and the regulation of the mitotic cell cycle. Concerning cellular components, the distinct genes were most abundant in areas related to the cytoskeleton and cell–cell junctions. Molecular functions revealed that the DEPs were primarily enriched in transcription factor binding, receptor signaling protein activity, ubiquitin binding, MAP kinase activity, and NF-κB binding, as depicted in Fig. 5A.

As shown in Fig. 5B, the KEGG signal pathway enrichment analysis indicated that DEPs were implicated in various pathways, including TGF-beta, WNT, thyroid hormone, VEGF, mTOR, NF-κB, PI3K-Akt, MAPK, and RAS signaling pathways. The outcomes of both GO functional biological function enrichment analysis and KEGG signaling pathway enrichment analysis highlighted those biological functions pertinent to thyroid cancer encompassed cell proliferation, cytoskeleton maintenance, and cell–cell junction regulation.

To identify the differentially expressed proteins strongly associated with IEFVPTC, we conducted a protein interaction network analysis based on the 181 differential proteins using NetworkAnalyst, as depicted in Fig. 5C. Only proteins ranking in the top for both degrees (the number of interactions of each node) and betweenness centrality (the degree of impact on interactions between other nodes in the network) parameters were acknowledged as hub genes. In conclusion, the hub genes encompassed *CUL1, PRKDC, DLG4, VAV2, FLNC, UBR5, MAST3, SRSF1,* and *SIN3B* (Supplementary Table 7).

### Trajectory inference of tumorigenesis of encapsulated follicular-pattern thyroid tumor

**Figure 6:**
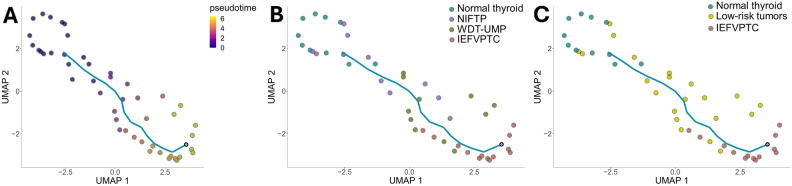
Pseudotime trajectory analysis for encapsulated follicular-patterned thyroid tumors. The cells are colored by pseudotime (**A**), subtypes of thyroid tumors (**B**), and group assignments of the tumors (**C**).

We utilized the Monocle3 workflow with the proteomics profile to elucidate the dynamic process patterns experienced by samples and subsequently organized these samples based on their progression through the process. In our study, we identified a single linear trajectory (Fig. 6A, B). The UMAP projection revealed trajectories that reflect a smooth progression, starting from normal thyroid tissue and developing towards WDT-UMP and NIFTP, eventually leading to IEFVPTC, which is considered malignancy.

## Discussion

Recently, LC–MS/MS has emerged as a valuable tool in enhancing our understanding of biology and diagnosis of thyroid neoplasms. For example, Huang et al., employed LC–MS/MS to investigate molecular distinctions among follicular thyroid adenoma, follicular thyroid carcinoma, and follicular variant of papillary thyroid carcinoma (FVPTC). They observed significant similarities between FTC and FVPTC, distinguishing them from FA. Furthermore, ANXA1 emerged as a promising biomarker for differentiating FVPTC from other thyroid tumors^13^. Yasemin et al., utilized Matrix-Assisted Laser Desorption/Ionization (MALDI) Mass Spectrometry Imaging (MSI) to differentiate NIFTP from normal thyroid parenchyma. Their findings revealed similar peptide profiles between NIFTP and encapsulated and infiltrative FV-PTC^14^. In 2022, Sun et al.^15^ presented a comprehensive spectral library for DIA and PRM analysis of thyroid tissue samples, identifying nine characteristic proteins capable of distinguishing between FA and FTC. Their data suggested altered ferroptosis pathways in malignant follicular carcinoma.

IEFVPTC is now recognized as a distinct entity rather than a subtype of PTC. It shares a *RAS*-like mutational and transcriptomic profile with FA and FTC, while classic PTC and the infiltrative follicular subtype of PTC exhibit *BRAF*V600E-like molecular profiles^3^. IEFVPTC displays morphological similarities with NIFTP and WDT-UMP, characterized by tumors surrounded by a fibrous capsule and nuclear features resembling papillary thyroid carcinoma. However, IEFVPTC can invade vessels in the capsule and develop distant metastasis^8^. Diagnosing and identifying these follicular-patterned thyroid nodules have posed significant challenges to pathologists. To date, there has been no comprehensive study exploring the proteomic profile and biological pathways of this encapsulated follicular-patterned tumors with nuclear features of PTC group. This research marked the first analysis of the proteomics profile in these tumors.

In our current study, we employed shotgun LC–MS/MS to compare the proteomic similarities and differences among IEFVPTC, NIFTP, and WDT-UMP. Our analysis identified 181 differentially regulated proteins in IEFVPTC compared to the others (Fig. 4A). Conversely, no differentially expressed proteins were found between NIFTP and WDT-UMP (Fig. 4B). On the PCA graph, IEFVPTC was distinctly separated from the other groups, while NIFTP was distributed in the same area as WDT-UMP. This suggests that NIFTP and WDT-UMP share significant similarity with each other but are distinct from IEFVPTC, despite exhibiting similar molecular alterations. According to the updated WHO classification, IEFVPTC is classified as malignant and subdivided into minimally invasive, encapsulated angioinvasive, and widely invasive categories^3^. Minimally invasive tumors are considered low-risk and may be treated with only local excision. While widely invasive or angioinvasive tumors may require completion thyroidectomy and additional therapy to prevent recurrence and/or distant metastasis, based on individual clinical risk assessment, such as a large tumor size (greater than 4 cm), extrathyroidal extension, or metastasis^16^. NIFTP and WDT-UMP are categorized as low-risk neoplasms, representing tumors that are borderline and possess characteristics intermediate between benign and malignant tumors. While these neoplasms have the potential to develop metastases, the occurrence of metastasis is exceptionally rare. Lobectomy and close monitoring for NIFTP/WDT-UMP patients are crucial to ensure that these tumors do not progress^16^. Hence, accurate diagnosis of IEFVPTC remains essential to avoid unnecessary or potentially harmful surgical interventions.

Jing et al.^17^ discovered that in FVPTC tissues, compared to classical PTC tissues, the genes ZMAT4, SLC5A8, DIO1, MT1H, and CUX2 showed the highest upregulation, while TMPRSS6, SFTPB, SYT12, SLC27A6, and TMPRSS4 were the most notably downregulated, at the mRNA level. Li et al.^18^ performed a differential analysis on FVPTC versus normal tissue focusing on miRNA expression. They identified that epigenetic changes involving five miRNAs (hsa-mir-222, hsa-mir-221, hsa-mir-34a, hsa-mir-214, and hsa-mir-138-2) could be influencing the expression of genes such as BCL2, BCL2L11, PEG3, ALDH1A1, PLA2R1, TFCP2L1, RAB23, TK1, and CTSB, which may play roles in the development of FVPTC. At the protein level, in a comparison of IEFVPTC to NIFTP/WDT-UMP, we found that six were upregulated (*NPAP1, STK33, O51I2, DLG4, THUM2, CODA1*), and four were downregulated (*CCHCR, STAR5, DDX6, LIGO2*). *STK33* plays a pivotal role in cancer cell proliferation and metastasis, mediates angiogenesis, TGFβ, and inflammatory response in esophageal squamous cell carcinoma^19^, promotes cell migration and invasion and suppress p53 gene expression in the large cell lung cancers^20^. *CCHCR1* is upregulated in cutaneous squamous cell cancer and associated with EGFR expression^21^. *DDX6* as oncogene in gastric cancer cells through promotion of c-Myc expression by association with the mRNA of c-Myc^22^. *LIGO2* is expressed in gastric cancer tissues, regulating cell motility, tumorigenesis, and angiogenesis^23^. While the roles of these top-ranked discriminatory proteins have been extensively studied in other diseases, limited evidence exists about their associations with malignant thyroid tumors. Therefore, we hypothesize that these costimulatory molecules may induce oncogenic effects in malignant thyroid tumors.

Through GO enrichment analysis, numerous biological processes were identified, offering potential insights into the mechanisms underlying IEFVPTC development. DEPs in IEFVPTC were found to be significantly involved in biological processes such as the cell cycle process, MAP kinase activity, NF-κB binding, DNA damage checkpoint, ubiquitin ligase complex, cell–cell, and cell-substrate junctions, as well as cytoskeletal components. Pathway analysis indicated differential expression of key proteins associated with cell migration, mapping to molecular pathways like actin cytoskeleton signaling and the remodeling of epithelial adherens junctions. Notably, these changes were similarly observed in the comparison of PTC and FTC^24^. KEGG enrichment analysis further highlighted the significance of DEPs in IEFVPTC, with enrichment in pathways such as ERBB, Hippo, TP53, TGF-beta, WNT, VEGF, mTOR, NF-κB, PI3K-Akt, TNF, MARK, RAS signaling pathways, thyroid hormone signaling pathway, and adherens junctions. These pathways are known to play roles in the multistep tumorigenic process of thyroid cancer^25^.

In the PPI network, we observed significant connectivity among various proteins, including *CUL1, PRKDC, DLG4, VAV2, FLNC, UBR5, MAST3, SRSF1,* and *SIN3B. CUL1*, known to regulate cell proliferation, cell cycle, migration, invasion, and metastasis, has been associated with poor prognosis in gastric cancer, colorectal cancer, melanoma, lung, and breast cancer^26,27^. The *PRKDC* gene encodes the DNA-dependent protein kinase catalytic subunit (DNA-PKcs), playing a crucial role in DNA double-strand break repair and immune tolerance regulation. Elevated DNA-PKcs activity during tumor development affects tumor suppressor gene regulation, contributing to malignant progression. *PRKDC* mutations are linked to higher tumor mutational burden and better respond to immune checkpoint inhibitors, suggesting its potential as a predictive biomarker for immunotherapy^28^. High levels of *VAV2* transcripts and their regulated gene signatures are associated with poor prognosis in head and neck squamous cell carcinoma^29^. *FLNC*, an actin cross-linking protein, is involved in cell morphology regulation and is a potential therapeutic target and biomarker for glioblastoma multiforme progression^30^. *UBR5* is implicated in the regulation of DNA damage response, metabolism, transcription, and apoptosis, serving as a key regulator in cancer biology^31^*. MAST3* is involved in promoting proliferation and inflammation in synovial cells by regulating NF-κB signaling pathway^32^. *SRSF1*, a member of the SR-protein family, is a potential oncoprotein involved in various biological functions, including alternative splicing regulation and tumorigenesis promotion in multiple cancers^33^. These hub genes identified in our study have implications for the response to immune checkpoint inhibitors therapy (*PRKDC*) and offer potential prognostic markers (*CUL1, VAV2)*, presenting a novel avenue or additional treament for thyroid cancer therapeutics.

Nikiforov et al.^34^ have proposed that NIFTP likely serves as the precursor of IEFVPTC. However, to date, there has been no study providing definitive evidence to support this assertion. Utilizing our molecular profile, we proposed a trajectory to recapitulate the progression sequence from NIFTP/WDT-UMP to IEFVPTC, demonstrating the potential for invasion (Supplementary Fig. 2). Consequently, we concur with the aforementioned statement that NIFTP/WDT-UMP or low-risk tumors may indeed serve as the “benign” counterparts to IEFVPTC (Fig. 6C).

Despite the insights gained from our current work, there are some limitations. Firstly, we utilized non-targeted proteomics testing to detect peptide profiles, lacking a comprehensive and integrated analysis incorporating multiple omic platforms. In our next phase, we aim to incorporate mRNA and DNA methylation profiles to enhance our understanding of the biological features of thyroid tumors. Additionally, this study represents our initial effort in identifying protein marker candidates for distinguishing follicular-pattern thyroid tumors. In subsequent steps, we plan to validate these biomarker candidates as immunohistochemistry (IHC) markers in a larger sample cohort from multiple clinical centers, including fine-needle aspiration (FNA) samples and formalin-fixed paraffin-embedded (FFPE) samples. Furthermore, other follicular-patterned thyroid neoplasms such as follicular adenomas (FA), follicular thyroid carcinomas (FTC), and thyroid nodules with different backgrounds like Hashimoto's thyroiditis and goiter were not analyzed in the current study, and these will be subjects of investigation in future studies. The distinct biological characteristics and prognoses of the IEFVPTC subclasses (minimally invasive, encapsulated angioinvasive, and widely invasive) were also not analyzed. Moreover, the study identified several hub genes but has yet to pinpoint the specific pathways related to tumorigenesis that could inform potential therapeutic approach for follicular thyroid tumors.

## Conclusion

To our knowledge, this study represents the initial proteomic analysis of follicular-pattern thyroid tumors aimed at identifying specific protein signatures capable of distinguishing IEFVPTC from low-risk tumors (NIFTP and WDT-UMP). These discoveries have the potential to offer valuable insights into tumor biology and serve as a basis for the development of novel therapeutic approaches for follicular-patterned thyroid neoplasms.

## Methods

### Thyroid tissue specimens

As shown in Table 1, 46 formalin-fixed paraffin-embedded (FFPE) specimens, specifically, 13 IEFVPTC, 11 NIFTP, 10 WDT-UMP and 12 thyroid normal specimens were obtained from Department of Pathology at King Chulalongkorn Memorial Hospital between January 1st, 2019 and September 30th, 2023, with approval from the Institutional Review Board of the Faculty Medicine, Chulalongkorn University, Bangkok, Thailand (approval no. COA.No. 1369/2023-IRB 0628/66). Two pathologists (T.PX.N and S.K.) independently confirmed the pathological diagnosis in the above tissues and had a consensus in accordance with the 5th World Health Organization Classification of Tumors of Endocrine Organs^3^. Each FFPE specimens was cut into 10 µm thick sections. After that, we isolated a tumor region from the FFPE section to ensure the samples contained at least 90% tumor cells and put into 3 clean Eppendorf tubes for triplicates. 138 tubes were analyzed by the shotgun LC–MS/MS method to investigate the protein alterations.

### Protein preparation and shotgun LC–MS/MS analysis

Total protein was extracted from FFPE specimens by using 0.5% SDS. After incubating at 50ºC for 60 min and centrifuge at 10,000 rpm for 30 min, the lower solution was carefully transferred into a new microtube. After protein quantitation (BCA method)^35^, five micrograms of protein samples were reduced with 5 mM dithiothreitol (DTT) in 10 mM AMBIC at 60 °C for 1 h, alkylated 15 mM iodoacetamide (IAA) in 10 mM AMBIC at room temperature for 45 min in the dark. Extracted proteins were digested by using porcine trypsin of sequencing grade (1:20 ratio) for 16 h at 37 °C. The tryptic proteins were dried using a speed vacuum concentrator and resuspended in 0.1% formic acid for nano-liquid chromatography tandem mass spectrometry (nanoLC-MS/MS) analysis.

LC–MS/MS data was acquired by an Ultimate3000 Nano/Capillary LC System (Thermo Scientific, UK) coupled to a Hybrid quadrupole Q-Tof impact II™ (Bruker Daltonics) equipped with a Nano-captive spray ion source. Briefly, one microlitre of peptide digests were enriched on a µ-Precolumn 300 µm i.d. X 5 mm C18 Pepmap 100, 5 µm, 100 A (Thermo Scientific, UK), separated on a 75 μm I.D. × 15 cm and packed with Acclaim PepMap RSLC C18, 2 μm, 100 Å, nanoViper (Thermo Scientific, UK). The C18 column was enclosed in a thermostatted column oven set to 60 °C. Solvent A and B containing 0.1% formic acid in water and 0.1% formic acid in 80% acetonitrile, respectively were supplied on the analytical column. A gradient of 5–55% solvent B was used to elute the protein at a constant flow rate of 0.30 μl/min for 30 min. Electrospray ionization was carried out at 1.6 kV using the CaptiveSpray. Nitrogen was used as a drying gas (flow rate about 50 l/h). Collision-induced-dissociation (CID) product ion mass spectra were obtained using nitrogen gas as the collision gas. Mass spectra (MS) and MS/MS spectra were obtained in the positive-ion mode at 2 Hz over the range (m/z) 150–2200. The collision energy was adjusted to 10 eV as a function of the m/z value. The LC–MS analysis of each sample was done in triplicate.

### LC–MS/MS data processing

Protein quantification in individual samples was conducted using MaxQuant 2.2.0.0 with the Andromeda search engine, correlating MS/MS spectra to the Uniprot *Homo sapiens* database^36^. Standard settings for label-free quantitation were applied, including a maximum of two missed cleavages, a mass tolerance of 0.6 dalton for the main search, trypsin as the digesting enzyme, fixed carbamidomethylation of cysteine, and variable modifications such as oxidation of methionine and acetylation of the protein N-terminus. Identified proteins required protein with a minimum length of 7 amino acids and at least one unique peptide. Further, only proteins with a minimum of two proteins, including at least one unique peptide, were considered for identification and subsequent data analysis. Protein false discovery rate (FDR) was set at 1%, estimated using reversed search sequences. The maximal number of modifications per peptide was set to 5. The *Homo sapiens* proteome from Uniprot served as the search FASTA file, with potential contaminants from MaxQuant’s contaminants.fasta file automatically included in the search space.

### Bioinformatical and statistical analysis

Data filtering, processing and statistical analysis of the MaxQuant output files was performed using R software programming language version 4.2.2. Data was filtered excluding the following hits: only identified by site, contaminants and reversed. For further analysis, a minimum threshold of 40% protein representation in each group is required for a presentation deemed suitable. The peptide intensity of each sample was median value from triplicate. We employed the DESeq2 pipeline to perform differential expression analysis^37^. Differentially expressed proteins in pairwise comparisons IEFVPTC-non IEFVPTC, NIFTP-non NIFTP, WDT-UMP-non WDT-UMP, IEFVPTC-NIFTP, IEFVPTC-WDT-UMP and NIFTP-WDT-UMP samples by two-sided t-test (adjusted p < 0.05) and fold change fc ≥ 2. The Benjamini-Hochberg (BH) multiple corrections were applied to reduce false-positive results. Venn diagrams was used to show the differences between protein lists originating from different differential analyses^38^. The heatmap was generated with the pheatmap R package (https://cran.r-project.org/web/packages/pheatmap/index.html), and the proteins in each row were subjected to unsupervised clustering.

Pathway enrichment analysis, protein–protein interaction (PPI) network construction and hub genes identification: Networkanalyst (https://www.networkanalyst.ca/) is a web-based application that allows users to visualize statistical meta-analysis, perform data mining, design biological networks, functional enrichment analysis (Gene Ontology -GO and Kyoto Encyclopedia of Genes and Genomes -KEGG pathway).

GO enrichment analysis is a statistical method used in bioinformatics to determine if a specific group of genes correlates with certain functional categories, aiding in the clarification of the associated biological processes. The GO system organizes these functions into three primary categories: molecular function, which describes the specific activities a gene's product performs at the molecular level; biological process, which highlights the broader cellular or physiological roles a gene plays in conjunction with other genes; and cellular component, which identifies the exact cellular location where the gene's product operates^39^.

KEGG pathway enrichment analysis is a technique in bioinformatics that helps interpret extensive biological data sets, such as lists of genes or proteins, by linking them to established biological pathways. This type of analysis is valuable for understanding the biological functions and interactions among different cellular or organismal components^40^.

Biological data integration was achieved using robust statistical procedures and the data were visualized using PPI networks. The significance of a node was determined by the number of proteins interacting with it. Nodes within the top 10% based on degree value were chosen, indicating potential physiological regulatory functions.

For clinicopathological analyses, the P values of the three groups' comparisons were calculated by one-way analysis of variance (ANOVA). All the hypothesis tests were considered significant when P < 0.05.

Trajectory analysis: We utilized the mococle3 workflow (https://cole-trapnell-lab.github.io/monocle3/docs/clustering/) to examine the developmental pathway undertaken by different subtypes of encapsulated follicular thyroid tumors as they progress through tumorigenesis^41^.

### Ethics declarations

The research was established according to the ethical guidelines of the Helsinki Declaration and was approved by the Institutional Review Board of Faculty of Medicine, Chulalongkorn University (COA.No. 1369/2023-IRB 0628/66). Written informed consent was obtained from all subjects and their legal guardians.

### Supplementary Information

Supplementary Information.

## Data Availability

All data generated or analyzed and its supplementary information files during this study are included in this published article. The mass spectrometry proteomics data have been deposited to the PRIDE Archive (http://www.ebi.ac.uk/pride/archive/) via the PRIDE partner repository with the data set identifier PXD053567.
