# Supplementary material for: Proteomics profile in encapsulated follicular patterned thyroid neoplasms
Source: Sci Rep. 2024 Jul 16;14:16343. doi: 10.1038/s41598-024-67079-6 (PMC11252349; doi:10.1038/s41598-024-67079-6)
Supplement: Supplementary file 1 — Supplementary Information. [file 41598_2024_67079_MOESM1_ESM.zip › Supplementary figure legends.docx]

**SUPPLEMENTARY FIGURE LEGENDS**

Supplementary Figure 1: Data quality analysis. (A) Identified protein numbers were consistent with each replicated run. (B) Spearman correlation of paired technical replicates.

Supplementary Figure 2: Putative Scheme of Follicular-Pattern Thyroid Carcinogenesis. IEFVPTC: Invasive Encapsulated Follicular Variant Papillary Thyroid Carcinoma, NIFTP: Non-invasive Follicular Thyroid Neoplasm with Papillary-like Nuclear Features, WDT-UMP: Well-Differentiated Tumor of Uncertain Malignant Potential
