# Supplementary figures and images for: Proteomics profile in encapsulated follicular patterned thyroid neoplasms
Source: Sci Rep. 2024 Jul 16;14:16343. doi: 10.1038/s41598-024-67079-6 (PMC11252349; doi:10.1038/s41598-024-67079-6)

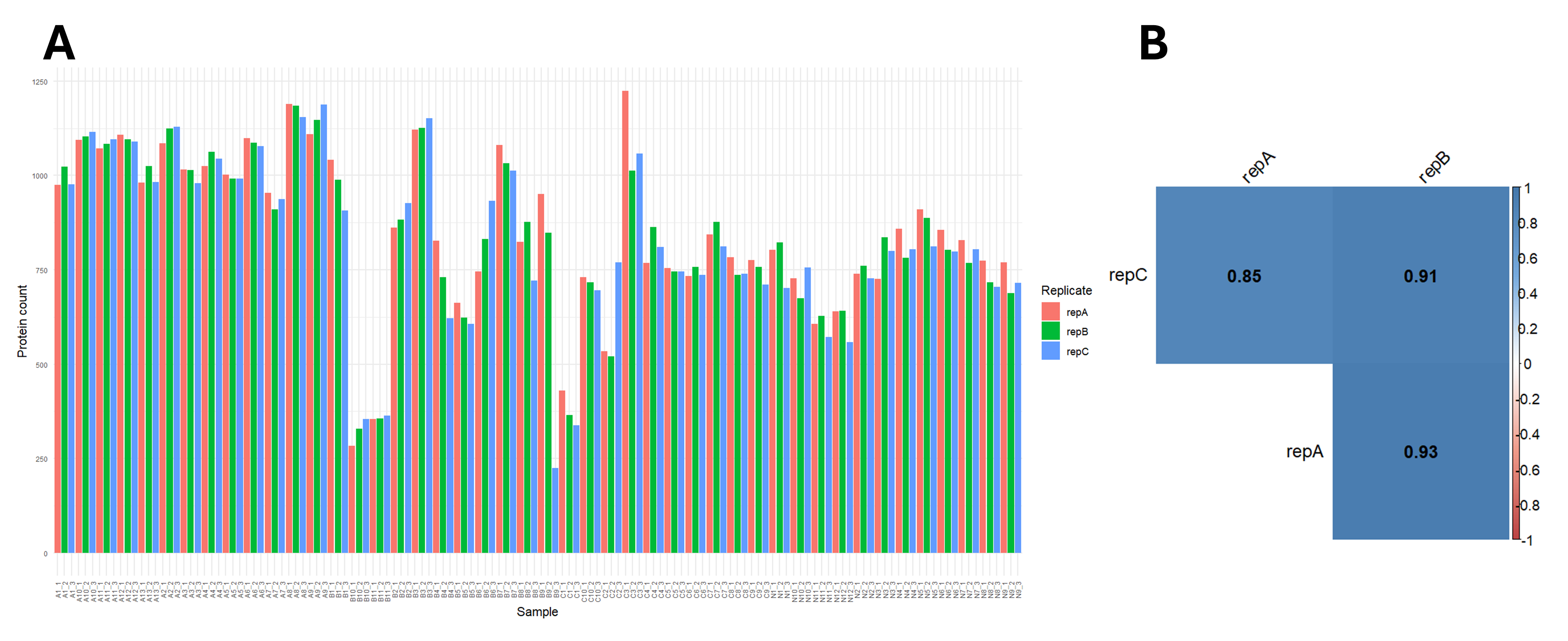

Supplement: Supplementary file 1 — Supplementary Information. [file 41598_2024_67079_MOESM1_ESM.zip › Fig 1S.tif]

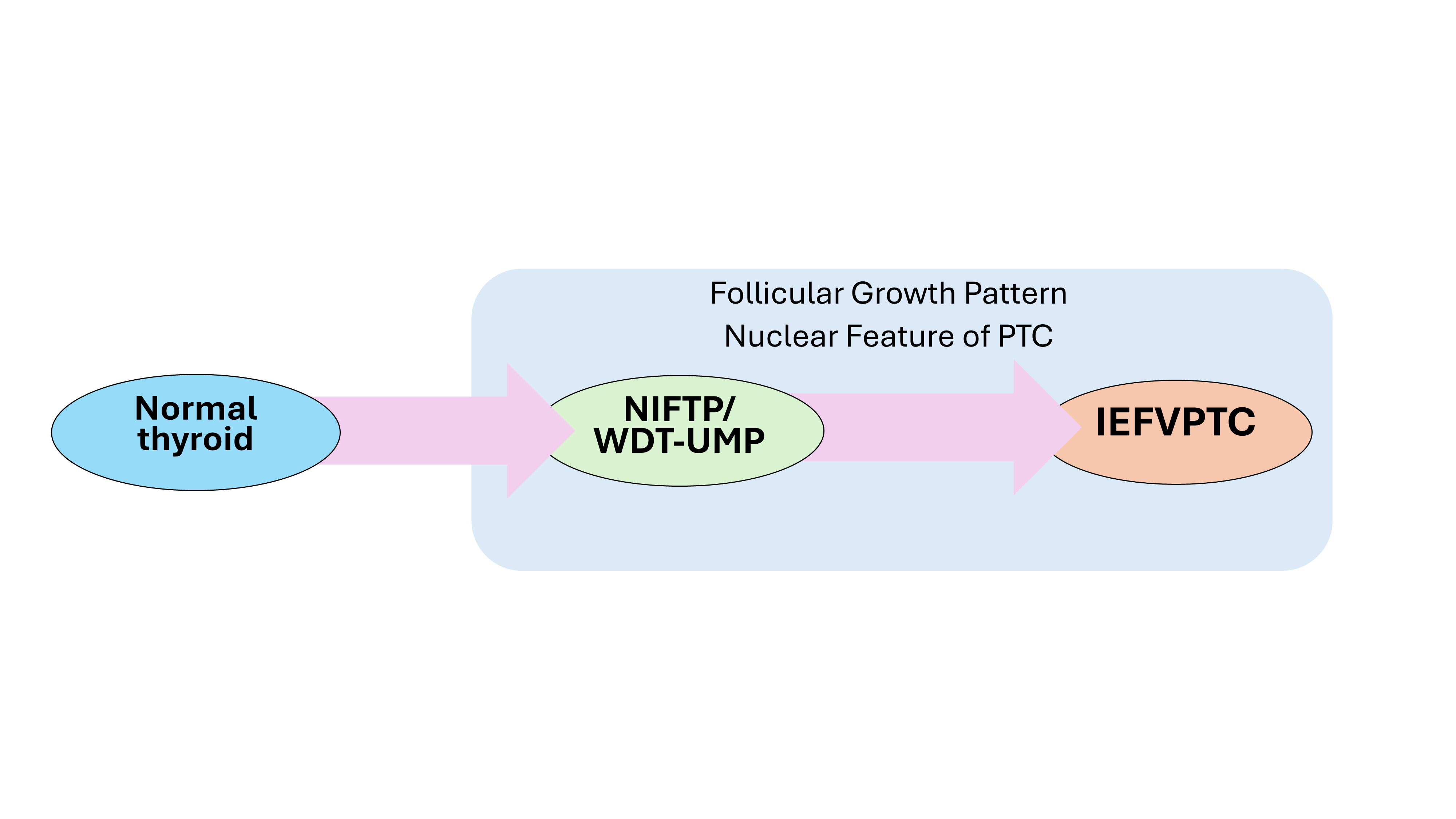

Supplement: Supplementary file 1 — Supplementary Information. [file 41598_2024_67079_MOESM1_ESM.zip › Fig 2S.tif]
